# Peptidoglycan Recognition Protein 3 Does Not Alter the Outcome of Pneumococcal Pneumonia in Mice

**DOI:** 10.3389/fmicb.2018.00103

**Published:** 2018-02-01

**Authors:** Anshu Shrivastav, Alexander N. Dabrowski, Claudia Conrad, Nelli Baal, Holger Hackstein, Stephanie Plog, Kristina Dietert, Achim D. Gruber, Philippe D. N’Guessan, Sahar Aly, Norbert Suttorp, Janine Zahlten

**Affiliations:** ^1^Department of Internal Medicine/Infectious Diseases and Pulmonary Medicine, Charité – Universitätsmedizin Berlin, Berlin, Germany; ^2^Institute for Clinical Immunology and Transfusion Medicine, Justus-Liebig-Universität Giessen, Giessen, Germany; ^3^Department of Veterinary Pathology, Freie Universität Berlin, Berlin, Germany

**Keywords:** innate immunity, *Streptococcus pneumoniae*, pneumococcal pneumonia, peptidoglycan recognition proteins, PGLYRP, PGRP

## Abstract

Pneumococci frequently cause community-acquired pneumonia, a disease with high mortality rates, particularly in young children and in the elderly. Endogenous antimicrobial peptides and proteins such as PGLYRP3 may contribute to the progression and outcome of this disease. Since increasing antibiotic resistant strains occur all over the world, these endogenous antimicrobial molecules are interesting new targets for future therapies. In this study, the expression pattern of PGLYRP3 was analyzed in alveolar epithelial cells, alveolar macrophages and neutrophils. Additionally, the function of PGLYRP3 during *Streptococcus pneumoniae*-induced pneumonia was investigated in a murine pneumococcal pneumonia model using PGLYRP3KO mice. PGLYRP3 is expressed in all selected cell types but pneumococcus-dependent induction of PGLYRP3 was observed only in neutrophils and alveolar macrophages. Interestingly, there were no significant differences in the bacterial loads within the lungs, the blood or the spleens, in the cytokine response, the composition of immune cells and the histopathology between wild type and PGLYRP3KO mice. Finally, we could neither observe significant differences in the clinical symptoms nor in the overall survival. Collectively, PGLYRP3 seems to be dispensable for the antibacterial defense during pneumococcal pneumonia.

## Introduction

*Streptococcus* (*S*.) *pneumoniae* was discovered as a major cause of pneumonia in 1881 AD. Nearly a century ago Sir William Osler declared the pneumococcus as ‘the captain of all the men of death’ (Watson et al., 1993). This still holds true, since pneumococcal pneumonia causes to this day approximately two million deaths globally per year and is found to especially affect immunocompromised patients, children younger than 5 or adults older than 65 years (Dockrell et al., 2012; Prina et al., 2015).

Emerging antibiotic resistant strains of *S. pneumoniae* and the ability of the bacteria to evade available vaccines (van der Poll and Opal, 2009) display the need to develop new prevention and/or treatment strategies. Endogenous antimicrobial substances could be an alternative to the current application of antibiotics.

Bactericidal or bacteriostatic molecules within the host such as antimicrobial peptides (AMPs) and peptidoglycan recognition proteins (PGRPs) are broadly expressed mainly on body surfaces to defend the host from invading pathogens. PGRPs have been discovered in most animals including insects, mollusks, echinoderms, and invertebrates but not in nematodes or in plants. Until now, about hundred PGRPs across the whole spectrum of organisms haven been identified (Dziarski and Gupta, 2006; Kurata, 2014). Most PGRPs contain a PGRP domain that is approximately 165 amino acids long and comparable to the bacteriophage and bacteria type 2 amidases. The PGRP domains between the species are almost 42% identical and about 55% similar (Dziarski and Gupta, 2006).

According to the present nomenclature, insect PGRPs have been designated as PGRPs while mammalian PGRPs have been termed as PGLYRPs. PGLYRPs can be separated into two groups, the first group are proteins with amidase activity and the second group is described to be bactericidal or bacteriostatic (Dziarski and Gupta, 2006).

Mammals express four different PGLYRPs. PGLYRP2 belongs to the amidase active PGLYRPs and PGLYRP1, PGLYRP3 and PGLYRP4 are described to be bactericidal or bacteriostatic (Dziarski and Gupta, 2006). This antibacterial activity includes several different Gram-positive and Gram-negative bacteria species such as *Staphylococcus aureus, Listeria monocytogenes, Bacillus subtilis, Escherichia coli, Streptococcus pyogenes, Salmonella enterica, Shigella sonnei, Proteus vulgaris, Pseudomonas aeruginosa*, and *Lactobacillus acidophilus* (Lu et al., 2006; Wang et al., 2007).

Murine PGLYRP3 is expressed in hematopoietic and epithelial cells and was also found in the lung (Mathur et al., 2004). The first indication for a potential role of PGLYRP3 in pulmonary infections was reported. In that study, mice were intranasally treated with recombinant human PGLYRP3 followed by an infection with *S. aureus*. The mice which were pretreated with rPGLYRP3 had a significantly lower bacterial load in their lungs compared to the control mice (Lu et al., 2006).

Other publications showed that PGLYRP3 expression is enhanced by bacteria, bacterial products or synthetic compounds in keratinocytes, oral and corneal epithelial cells which is likely mediated by pattern recognition receptors (PRRs) such as Toll like receptors (TLRs) and Nod like receptors (NLRs) (Uehara et al., 2005; Lu et al., 2006; Ma et al., 2010).

More recent research indicates that mammalian PGLYRPs may also have immunomodulatory functions which are independent of their bactericidal or enzymatic properties (Dziarski and Gupta, 2010). These immune modulatory functions were suggested to be even more important than direct antibacterial effects of these proteins, because (i) the direct antimicrobial functions are often inhibited by physiological concentrations of, e.g., salt and proteins, (ii) the relatively high dosage needed for antimicrobial activity is often not reached in the *in vivo* situation and (iii) immune modulation and antimicrobial functions can be mediated *via* independent mechanisms as seen for PGLYRPs where non-bactericidal immune modulatory PGLYRPs were expressed by *E. coli* (De Marzi et al., 2015; Dukhanina et al., 2015; Hancock et al., 2016). For example, PGLYRP3 enhances phagocytosis and the pro-inflammatory response to peptidoglycan (PGN) in THP-1 cells (De Marzi et al., 2015). In contrast to that, a knockdown of PGLYRP3 in an intestinal epithelial cell line (CaCo2) enhanced the expression of PGN-induced inflammatory cytokine response, whereas an overexpression of PGLYRP3 caused a downregulation of the cytokine expression (Zenhom et al., 2011). In another study, PGLYRP3 protected the colon of wild type (wt) mice from a dextran sulfate sodium (DSS)-dependent inflammatory response and stabilized the barrier function of the intestinal epithelium by upholding the normal gut microbiota and inhibited the induction of IFN-γ in response to injury (Saha et al., 2010). Therefore, there seems to be a divalent pro- and anti-inflammatory function of PGLYRP3 depending on stimuli or cell type.

Because there is an urgent need of new alternatives for treating pneumococcal infections and PGLYRPs were shown to be active against a variety of Gram-positive and Gram-negative bacteria, but have never been tested for activity against *S. pneumoniae*, we wanted to address this question. Unfortunately, to the best of our knowledge, recombinant murine PGLYRP3 is not yet commercially available and data on protein expression and purification have not been published. Therefore, we aimed in this study to investigate whether PGLYRP3 can modulate the development and the outcome of pneumococcal pneumonia in a murine pneumococcal pneumonia mouse model using PGLYRP3KO mice.

## Materials and Methods

### Ethics Statement

Animal housing and experimental procedures complied with the Federation of European Laboratory Animal Science Associations (FELASA) guidelines and recommendations for the care and use of laboratory animals. The animal procedures for the *in vivo* experiments were reviewed and approved by the local institutional (Charité – Universitätsmedizin Berlin) and governmental (Landesamt für Gesundheit und Soziales Berlin, approval ID: G0251/12) authorities. All experiments were conducted under intraperitoneal (i.p.) anesthesia with ketamine and xylazine and suffering was minimized in compliance with the 3R principles.

### Animals

Breeding pairs of PGLYRP3KO as well as the breeding pairs of the corresponding wt mice (both on BALB/c background) were kindly provided by Prof. Dr. Roman Dziarski (Department of Microbiology and Immunology, Indiana University School of Medicine, Indianapolis, IN, United States). The breeding of the mice occurred in the central breeding facility of the Charité – Universitätsmedizin Berlin (Forschungseinrichtungen für Experimentelle Medizin, FEM). PGLYRP3KO mice were routinely genotyped upon subsequent breeding to ensure that they were homozygous for the PGLYRP3KO.

Fifteen male and 118 female BALB/c mice were sex-, age-, and weight-matched and used for the subsequent experiments. Female mice, 8–12 weeks old, were used for the *in vivo* experiments as follows: eight animals per group and 32 mice in total for ELISA experiments; flow cytometry data was acquired from the same animals which were used for the ELISA experiments; 22 animals, 11 per group were used for the determining the bacterial load in the lungs, spleens and blood; 56 animals, 26 per group were used for the survival experiments, in which one wt mouse died during the infection (anesthetic complication) and is therefore not included in the analysis; eight mice, with four mice in each group were used for histological analyses. The male mice, aged 4–6 months, were used for the primary cell isolation procedures.

### Genotyping PCR

Tail tips were incubated in lysis buffer (0.1 M Tris, 5 mM EDTA, 200 mM NaCl, 0.2% SDS) and with 30 μl of Proteinase K (10 mg/ml; Sigma–Aldrich, Darmstadt, Germany) overnight at 54°C. The next day, samples were centrifuged at 4,000 ×*g* for 10 min. The supernatants were mixed with 500 μl of isopropanol (Sigma–Aldrich, Darmstadt, Germany) for DNA precipitation. Samples were centrifuged (16,000 ×*g*, 5 min) and washed with 70% ethanol (Roth, Karlsruhe, Germany) two times, then the pellet was air-dried and the DNA was dissolved at 56°C for 30 min in 10 μl of nuclease free water.

Fifty nanograms of DNA in 50 μl reaction mix (5 μl of 10x DreamTaq^TM^ Green Buffer, 5 μl of 100 mM dNTP mix, 0.1 μM forward primer (either wt: GATAACCAAGATTTCTGCGACATTGC or PGLYRP3KO: TGCGAGGCCAGAGGCCACTTGTGTAGC), 0.1 μM reverse primer (common: TTCTGCAGCACTCTCTCCTCCATAG) (TIB MOLBIOL GmbH, Berlin, Germany), 1.25 μl of DreamTaq^TM^ DNA Polymerase) were amplified with the following cycling conditions: 3 min at 95°C, 30 cycles with 30 s at 95°C, 30 s at 64°C and 2 min at 72°C and a final step of 7 min at 72°C. PCR products were run on a 1% agarose gel (Promega, Mannheim, Germany) with 0.04% ethidium bromide (Invitrogen, Waltham, MA, United States). The bands were visualized under UV-light.

### Isolation of Primary Cells

Untreated wt mice were used for all *in vitro* methods. The mice were anesthetized i.p. with ketamine and xylazine (both Rotexmedica, Luitré, France), the abdominal cavity was opened and the mice were exsanguinated *via* the caudal vena cava. For the isolation of alveolar epithelial cells (AECs) heparin (Rotexmedica, Luitré, France) was injected into the heart before exsanguination.

#### Isolation of Alveolar Macrophages (AMs)

AMs were isolated as described previously (Koppe et al., 2012a). Briefly, the lungs were lavaged multiple times with 2 mM EDTA PBS (Gibco, Cambridge, MA, United States) (final volume 5 ml). The lavages were centrifuged (300 ×*g*, 10 min, 4°C) and the cell pellets were resuspended in 1 ml of RPMI1640 (Gibco, Cambridge, MA, United States) with 10% FCS (GE Healthcare, Little Chalfant, GB), 1% glutamine (Glu) and 1% penicillin/streptomycin (P/S) (both Sigma–Aldrich, Darmstadt, Germany). The AMs were seeded into cell culture plates and incubated at 37°C for 2 h. Then the medium was changed and cells were incubated overnight. Two hours before stimulation, medium was changed again to RPMI1640 with 2% FCS and 1% Glu.

#### Isolation of Alveolar Epithelial Cells (AECs)

Alveolar epithelial cells were isolated as described previously (Koppe et al., 2012a). Briefly, the lungs were perfused with 10 ml PBS *via* the right ventricle of the heart to remove the intravascular pool of cells. Two milliliters of Dispase (5000 U) (BD Biosciences, Heidelberg, Germany) and 0.5 ml of low melt agar (Invitrogen, Boston, MA, United States) were supplied into the lung. Once the agar had solidified the lungs along with the trachea were taken out and washed with PBS. Thymus and heart were removed, the lungs were incubated with 2 ml Dispase (37°C, 6 min) and transferred into petri dishes with 10 ml of DMEM (Gibco, Cambridge, MA, United States) supplemented with 1% P/S, 1% Glu, 25 mM HEPES and 0.1 mg/ml DNase I (Roche, Mannheim, Germany). The macerated lungs were passed through different cell strainer (100 μm, 70 μm, and 30 μm) to remove debris. 20 ml of DMEM with 10% FCS were added then centrifuged (200 ×*g*, 10 min, 4°C) and the pellet was resuspended in 1 ml of MACS buffer (PBS with 2 mM EDTA and 0.5% BSA). The AECs were collected by negative selection using the following antibodies: CD45, CD31, CD16/32, all biotinylated (BD Biosciences, Heidelberg, Germany). The MACS procedure was performed following manufacturer’s instructions (Miltenyi Biotec GmbH; Bergisch Gladbach, Germany). The AECs were centrifuged (200 ×*g*, 10 min, 4°C), resuspended in DMEM (1% P/S, 1% Glu, 25 mM HEPES, 10% FCS), seeded into appropriate cell culture plates and incubated at 37°C overnight. Two hours before stimulation, medium was changed again to DMEM with 2% FCS and 1% Glu.

#### Isolation of Neutrophils (PMNs)

The femurs and tibias were isolated and the soft tissue was removed. The ends of the bones were opened and the bone marrow were flushed out with sterile PBS. Afterward, cells were isolated as described elsewhere (Swamydas et al., 2015). Briefly, the cells were centrifuged (300 ×*g*, 10 min, 4°C), resuspended in 2 ml of MACS buffer and passed through a 30 μm cell strainer to remove any debris. The PMNs were separated by positive selection using the MACS mouse Anti-Ly-6G Microbead Kit (Miltenyi Biotech, Bergisch Gladbach, Germany) following the manufacturer’s instructions. After positive selection the neutrophils were resuspended in RPMI1640 with 2% FCS, 1% Glu and cells were stimulated immediately following isolation.

### Bacterial Strains

For *in vivo* infections and *in vitro* stimulation wt *S. pneumoniae* type 3 strain (NCTC 7978) was used. The bacteria were streaked out on Columbia agar plates with 5% sheep blood and incubated overnight at 37°C with 5% CO_2_. Single colonies were inoculated in Todd–Hewitt broth supplemented with 0.5% yeast extract (both BD Biosciences, Heidelberg, Germany) and incubated at 37°C with 5% CO_2_ till the pneumococci reached their mid-logarithmic phase (A600 0.3–0.4). The bacteria were harvested by centrifugation (1,800 ×*g*, 10 min) and resuspending in cell culture medium without antibiotics (*in vitro* stimulation) or in sterile PBS (*in vivo* infection) to reach the appropriate dosage.

### Quantitative Real-Time-PCR

RNA from primary cells was isolated using TRIzol (Life Technologies GmbH). One microgram of total RNA was used to transcribe cDNA (High-Capacity cDNA Reverse Transcription Kit; Life Technologies GmbH; Darmstadt; Germany). After pre-amplification (TaqMan^®^ PreAmp Master Mix Kit; Life Technologies GmbH) the cDNA was diluted and used for qPCR (TaqMan^®^ Gene Expression Master Mix and TaqMan^®^ Gene Expression Assays for GAPDH: Mm99999915_g1 and PGLYRP3: Mm01219437_m1; both Life Technologies GmbH). The protocol was performed according to the manufacturer’s instructions. Cycling conditions: 2 min, 50°C; 10 min, 95°C and 40 cycles with 15 s, 95°C and 1 min, 60°C. During preliminary testing we analyzed cytokine expression after infection with *S. pneumoniae* (MOI 1) of wt AECs and AMs at different time points (4, 8, 12, and 16 h, data not shown). These analyses indicated that the cytokines were most strongly induced between the 4 and 8 h time points. For best comparisons between all assayed targets and cell types, we routinely used these conditions (MOI 1, 6 h infection) for gene expression analyses.

### Mouse Infection

We used an infection dose comparable with other studies of intranasal serotype 3 *S. pneumoniae* infection in BALB/c mice (Khan et al., 2006, 2009; Seyoum et al., 2011; Sanfilippo et al., 2015). Therefore, female mice (8–12 weeks, 18–21 g) were anesthetized by i.p. injection of ketamine and xylazine and inoculated intranasally with 20 μl of bacterial suspension of *S. pneumoniae* (NCTC 7978) or 20 μl of sterile PBS as control (Schmeck et al., 2004). The animals were kept on a 12 h light/dark cycle, given free access to food and water and were monitored every 12 h to assess clinical manifestations such as appearance, behavior, grooming, respiration, body weight, and rectal temperature. Humane endpoints were defined as body temperature < 30°C, body weight loss ≥ 20%, cumbersome or strong pumping breathing, staggering and visible signs of pain. Animals were killed immediately after recognizing these symptoms by i.p. injection of ketamine and xylazine followed by cervical dislocation. In all experiments except the survival experiment (**Figure 6**), animals did not reach the predefined humane endpoints. No animal died by itself.

### ELISA

The mouse lungs were removed 48 h post infection, homogenized in sterile PBS at 4°C and passed through a 100 μm cell strainer, centrifuged (1,000 ×*g*, 10 min) and the supernatants were used to measure the cytokines. All ELISAs were performed according to the manufacturer’s instructions (KC from R&D Systems, Abingdon, United Kingdom; IL-1β, TNF-α, IL-6, and IL-10 from eBioscience, Frankfurt am Main, Germany).

### Bacterial Load

The bacterial load was calculated from the lung and spleen homogenates and EDTA blood of the mice infected with *S. pneumoniae* (NCTC 7978) 48 h after infection. Serial dilutions of the tissue homogenates and the blood were plated on Columbia agar with 5% sheep blood and incubated overnight at 37°C with 5% CO_2_. Then the CFUs were counted and the bacterial load was determined.

### Flow Cytometry

The lungs, infected with *S. pneumoniae* NCTC 7978) or PBS for 48 h, were perfused with HBSS (Gibco, Cambridge, MA, United States) and minced in RPMI1640 with 10% FCS. The digestion was performed by adding 0.15 U/ml type A collagenase and 9 U/ml DNase I (both Roche, Mannheim, Germany) at 37°C for 1 h. The single cell suspension was prepared by shearing the tissue with a 20G1aaa cannula and by passing the cell suspension through a 70 μm cell strainer. The cells were washed with HBSS, centrifuged (500 ×*g*, 7 min, 4°C) and blocked with 10% mouse serum in HBSS for 15 min and at 4°C. Cellular phenotyping was performed on a FACSAria III flow cytometer (BD Biosciences, Heidelberg, Germany). Differentially conjugated monoclonal antibodies against the following markers, as well as the appropriate isotype controls, were used for surface staining according to the manufacturer’s instructions: CD3e, CD4, CD8a, CD11b, CD11c, CD19, CD45, TCR-γδ, GR1, MHC II, CD49b, and SiglecF (BioLegend, Aachen, Germany). After the surface staining (30 min, 4°C, in the dark) cells were washed with HBSS, centrifuged (500 ×*g*, 7 min, 4°C), fixed by the addition of 2% PFA (Merck, Darmstadt, Germany) in PBS and incubated for 15 min on ice in the dark. The cells were washed again and the pellet was resuspended in HBSS. More than 200,000 flow cytometric events were acquired per sample. Absolute cell count was obtained using the trucount method (BD Biosciences).

Neutrophils were identified as CD45^+^, GR1^high^, CD11b^high^, AMs as CD45^+^, CD11c^high^, SiglecF^high^ and dendritic cells (DCs) as CD45^+^, SiglecF^-^ CD11c^high^, CD49b^-^, MHC II^high/int^. Classical B cells were identified as CD45^+^, CD3^-^, CD49b^-^, CD19^+^, whereas T cell populations were divided into T helper cells (CD45^+^, CD3^+^, CD4^+^, CD8^-^) and cytotoxic T cells (CD45^+^, CD3^+^, CD8^+^, CD4^-^). Furthermore, we stained for the two major innate lymphocyte subsets γδ-T cells (CD45^+^, CD3^+^, CD4^-^, CD8^-^, TCR-γδ^+^) and natural killer cells (CD45^+^, CD3^-^, CD49b^+^, CD19^-^). Because the classical NK cell marker NK1.1 is not expressed in BALB/c mice, we used the pan-NK cell marker CD49b in these experiments (Hackstein et al., 2012).

### Histology of the Lung

Mice were anesthetized 48 h p.i. with ketamine and xylazine then heparinized and exsanguinated. After ligation of the trachea, mouse lungs were carefully removed and immediately immersion fixed in 4% buffered formalin (Sigma–Aldrich, Darmstadt, Germany) at pH 7 for up to 48 h, embedded in paraffin, cut into 2-μm-thick sections and stained with hematoxylin and eosin (HE) after dewaxing in xylene and rehydration in decreasing concentrations of ethanol. Three evenly distributed sections per lung were microscopically evaluated to assess dissemination and quality of pathologic alterations.

### Digital Image Analysis

Hematoxylin and eosin stained slides were automatically digitalized by an Aperio CS2 (Leica Biosystems Imaging Inc., Vista, CA, United States) pathology scanner and lung areas were manually annotated to exclude adjacent tissues such as mediastinal adipose or lymphoid tissue from the analysis. To determine affected lung areas an adapted Genie histology pattern recognition algorithm (Leica Biosystems Imaging Inc.) was used.

### Data Analysis

**FIGURE 1:**
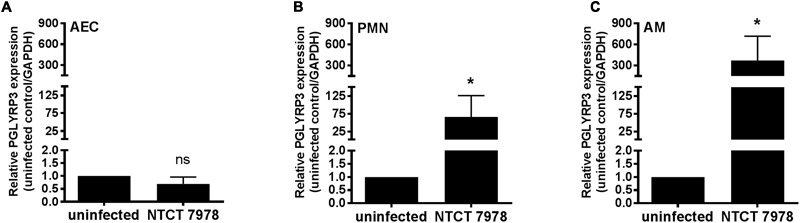
Pneumococcus-dependent expression of PGLYRP3 in primary cells. The PGLYRP3 expression in isolated primary mouse cells from wt mice was obtained by qPCR. Six hours after *in vitro* stimulation with *S. pneumoniae* (NCTC 7978; MOI 1) PGLYRP3 expression was unchanged in alveolar epithelial cells (AECs) **(A)**, but induced in bone marrow-derived neutrophils (PMNs) **(B)** and alveolar macrophages (AMs) **(C)**. Values are given as mean ± SEM (*n* = 3 for AECs and *n* = 4 for AMs and PMNs); Mann–Whitney *U* test: ^∗^*p* < 0.001.

**FIGURE 2:**
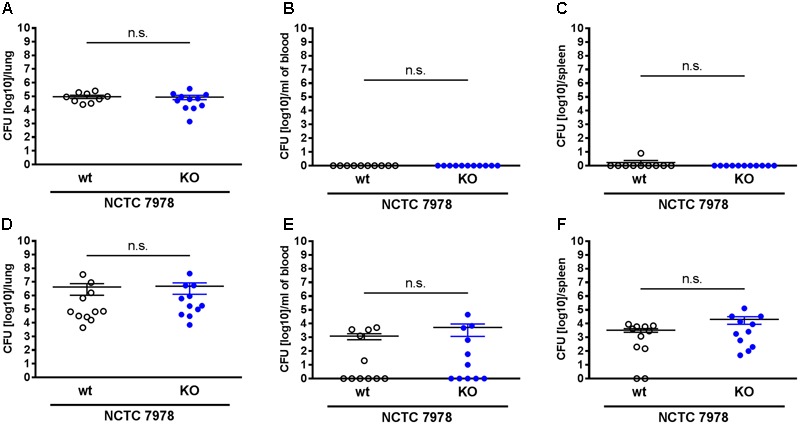
The loss of PGLYRP3 did not affect the bacterial load. Mice were infected intranasally with 10^5^ CFUs of the *S*. *pneumoniae* strain NCTC 7978 per mouse. The lungs **(A,D)**, the blood **(B,E)** and the spleens **(C,F)** were analyzed 12 h **(A–C)** and 48 h **(D–F)** after infection for the bacterial load in wt and PGLYRP3KO mice. Ten to eleven animals per group (mean ± SEM); Mann–Whitney *U* test: ns, not significant.

**FIGURE 3:**
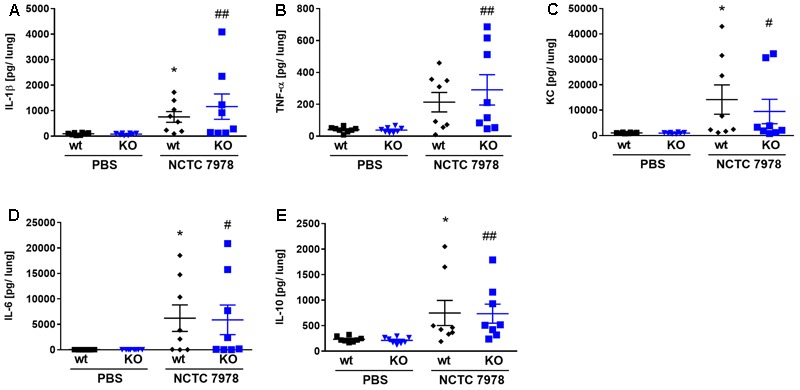
Cytokine response in the lung of wt and PGLYRP3KO mice. The *S. pneumoniae*-dependent cytokine response was analyzed 48 h post infection with 10^5^ CFUs of wt pneumococcal strain NCTC 7978 per mouse in lung homogenates. The production of IL-1β **(A)**, TNF-α **(B)**, KC **(C)**, IL-6 **(D)**, and IL-10 **(E)** was quantified by ELISA. Eight animals per group (mean ± SEM); Kruskal–Wallis with Dunn’s multiple comparison test was used for statistics. Significant differences in the cytokine response between uninfected and infected wt lungs were indicated as ^∗^*p* < 0.05 and the cytokine response in uninfected *vs.* infected PGLYRP3KO lungs as ^#^*p* < 0.05; ^##^*p* < 0.01.

**FIGURE 4:**
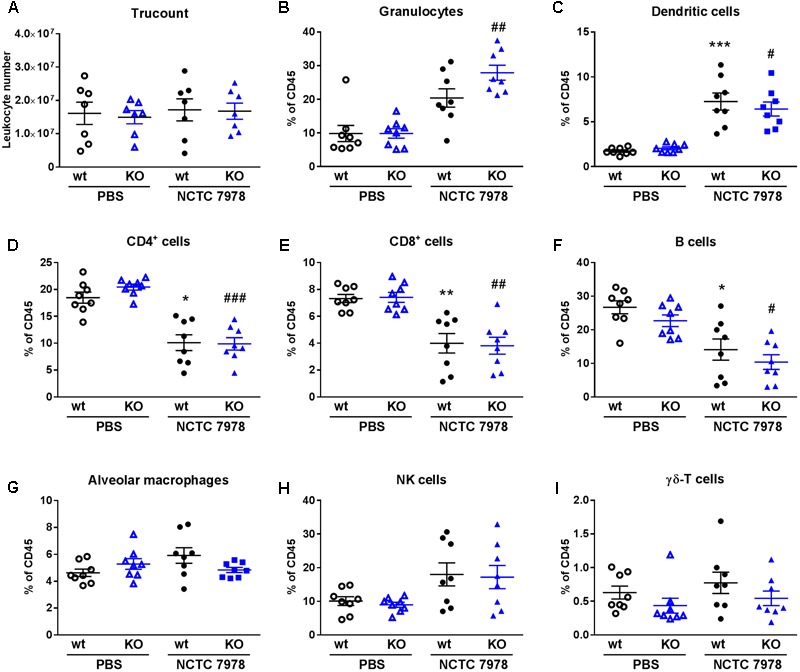
Comparison of the cell population in the lung. BALB/c wt and PGLYRP3KO mice were intranasally infected with 10^5^ CFUs *S. pneumoniae* (NCTC 7978) per mouse for 48 h. The amount of total leukocyte numbers and different CD45^+^ cell populations were analyzed by flow cytometry in whole lung homogenates. The numbers of leukocytes do not vary between the different conditions **(A)**. The proportional amount out of CD45^+^ cells (% of CD45) were shown for granulocytes **(B)**, DCs **(C)**, CD4^+^ T cells **(D)**, CD8^+^ T cells **(E)**, B cells **(F)**, AMs **(G)**, NK cells **(H)** and γδ-T cells **(I)**. Eight animals per group (mean ± SEM); Kruskal–Wallis with Dunn’s multiple comparison test: uninfected *vs.* infected wt: ^∗^*p* < 0.05; ^∗∗^*p* < 0.01; ^∗∗∗^*p* < 0.001 and uninfected *vs.* infected PGLYRP3KO: ^#^*p* < 0.05; ^##^*p* < 0.01; ^###^*p* < 0.001.

**FIGURE 5:**
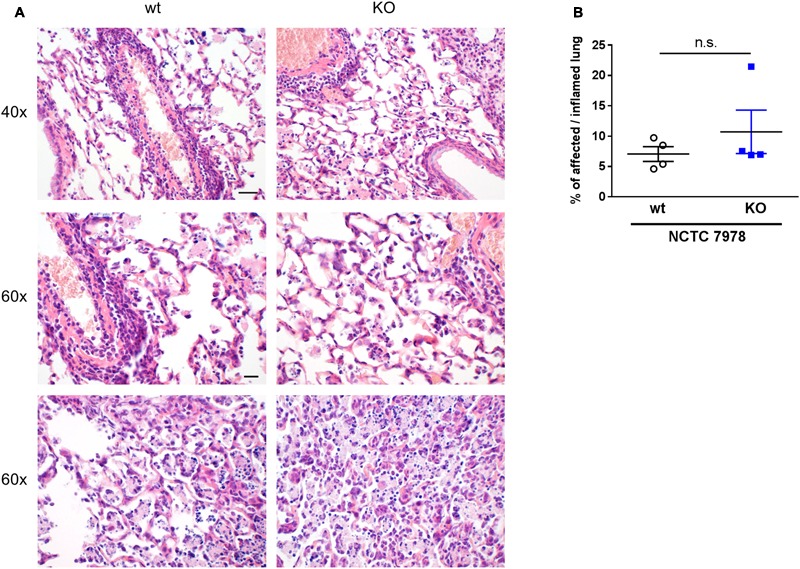
Histology and digital image analysis of infected wt and PGLYRP3KO mouse lungs. Lungs were harvested 48 h post infection with 10^5^ CFUs *S. pneumoniae* (NCTC 7978) per mouse, fixed in formalin and embedded in paraffin. Two micrometer sections were prepared and stained with hematoxylin and eosin **(A)** for histopathological analysis. Representative images for wt and PGLYRP3KO are shown (*n* = 4 per group). Bar (40×) 100 μm, Bar (60×) 20 μm. Quantitative image analysis on digitalized whole lung sections was performed. Genie pattern recognition algorithm was adapted and the percentages of affected/inflamed lung areas were assessed **(B)**. Four animals per group (mean ± SEM); Mann–Whitney *U* test was used for statistics: ns, not significant.

**FIGURE 6:**
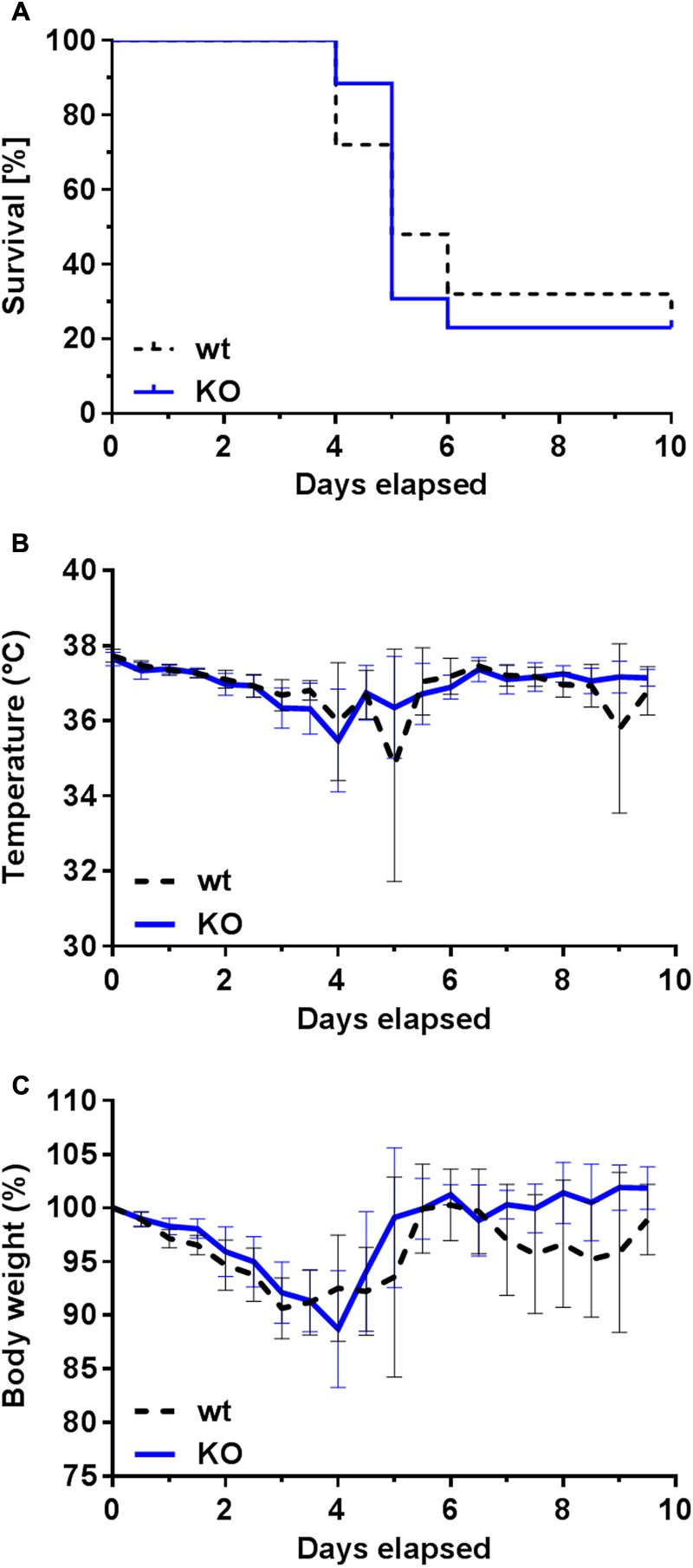
Survival rate and clinical signs of infected wt and PGLYRP3KO mice. Female BALB/c mice were intranasally infected with 10^5^ CFUs *S. pneumoniae* (NCTC 7978) per mouse. The survival was recorded every 12 h for 10 days. There was no significant difference observed between the two groups **(A)**. In total 25 wt and 26 PGLYRP3KO mice were used. Statistical analysis was done by log rank (Mantel–Cox) test. Every 12 h the clinical symptoms including temperature in °C (mean ± 95% CI) **(B)** and body weight shown in % of initial body weight (mean ± 95% CI) **(C)** were recorded in these mice. There were no differences between wt and PGLYRP3KO mice detectable neither in the progress of the temperature curve nor in the body weight loss.

Statistical analysis was done using Prism 6, GraphPad software, San Diego, CA, United States. The Mann–Whitney *U* test for comparing two populations (**Figures 1, 2, 5**) or Kruskal–Wallis with Dunn’s multiple comparison test was used when comparing more than two populations (**Figures 3, 4** and Supplementary Figures 1, 2). The Mantel–Cox log rank test was used to compare the mouse groups in the survival experiment (**Figure 6A**) and linear regression and correlation was used for **Figures 6B,C**.

## Results

### PGLYRP3 Is Induced in Neutrophils and Alveolar Macrophages by *S. pneumoniae*

It was demonstrated previously that PGLYRP3 is expressed in the lung (Mathur et al., 2004) but to our best knowledge, it is not known which cell types express PGLYRP3 in the lung or if PGLYRP3 might be induced upon stimulation with *S. pneumoniae*. The expression pattern of PGLYRP3 was analyzed *in vitro* in primary mouse cells 6 h post infection with *S. pneumoniae* (NCTC 7978) with a multiplicity of infection (MOI) of 1 and were compared to the uninfected control. The relative expressions of PGLYRP3 was determined in AECs, AMs, and bone marrow derived neutrophils (PMNs) from wt BALB/c mice. PGLYRP3 was found to be expressed in all tested cell types (**Figures 1A–C**). Upon infection with pneumococci no regulation of PGLYRP3 was measured in AECs (**Figure 1A**), but an approximately 66- and 373-fold increase of PGLYRP3 was observed in PMNs (**Figure 1B**) and AMs (**Figure 1C**), respectively.

### PGLYRP3 Seems Not to Be Bactericidal against *S. pneumoniae in Vivo*

To analyze the bacterial clearance in wt and PGLYRP3KO animals, the mice were intranasally infected with 10^5^ CFUs/mouse of *S. pneumoniae* (NCTC 7978). Mice were euthanized 12 or 48 h post infection and the CFUs in the lungs, the blood and the spleens were calculated. It has been previously published that PGLYRP3 has an antibacterial function against various Gram-positive and Gram-negative bacteria and recombinant PGLYRP3 protected mice from *S. aureus*-induced lung infection (Lu et al., 2006). Therefore, it was expected that the absence of PGLYRP3 would increase the bacterial load in *S. pneumoniae* infected mice. However, in our pneumonia mouse model we observed no differences between the CFUs isolated from the lungs (**Figures 2A,D**) of wt and PGLYRP3KO mice. At 12 h post infection, animals did not show bacteremia (**Figure 2B**) and only one wt mouse showed bacteria in the spleen (**Figure 2C**). There was an approximately 0.5 log scale higher bacterial load in the blood (**Figure 2E**) and approximately 1 log more bacteria found in the spleens (**Figure 2F**) of 48 h infected PGLYRP3KO mice in comparison with wt mice, but these observed differences did not reach statistical significance. Additionally, we observed that about half of the animals were bacteremic. When analyzed separately, bacteremic mice showed approximately 2 log higher bacterial loads in the lungs (Supplementary Figure 1A), which was significant for wt mice, but no difference was observed for bacterial burden in the spleen (Supplementary Figure 1C). No significant differences were seen between bacteremic or non-bacteremic wt and PGLYRP3KO mice (Supplementary Figures 1A–C).

### The Loss of PGLYRP3 Did Not Alter the Pneumococcus-Induced Cytokine Response in the Lung

There are recent reports that PGLYRP3 has, beside its antibacterial additional anti-inflammatory effects (Zenhom et al., 2011). Thus, we examined the pneumococcus-dependent (proinflammatory) cytokine response in the lung 48 h after the infection with 10^5^ CFUs of *S. pneumoniae* per mouse. Lung homogenates of uninfected and infected wt and PGLYRP3KO mice were analyzed for IL-1β (**Figure 3A**), TNF-α (**Figure 3B**), KC (**Figure 3C**), IL-6 (**Figure 3D**), and IL-10 (**Figure 3E**). No differences were found when comparing the lung cytokine levels of uninfected wt and PGLYRP3KO mice (**Figures 3A–E**). This was also true comparing infected wt and PGLYRP3KO mice (**Figures 3A,C–E**), with the exemption of a trending higher inflammatory response in infected PGLYRP3KO compared to the wt lung homogenates for TNF-α (**Figure 3B**). Nevertheless, we saw a significant increase between uninfected and infected wt mice for IL-1β (**Figure 3A**), KC (**Figure 3C**), IL-6 (**Figure 3D**), and IL-10 (**Figure 3E**). For uninfected *vs.* infected PGLYRP3KO mice IL-1β (**Figure 3A**), TNF-α (**Figure 3B**), KC (**Figure 3C**), IL-6 (**Figure 3D**), and IL-10 (**Figure 3E**) showed significant elevated levels. As done for bacterial burden, we also analyzed bacteremic and non-bacteremic mice separately. In general, there were no differences in the amount of cytokines between non-bacteremic and PBS-treated mice (Supplementary Figures 2F–J) and there were no significant differences between infected wt and PGLYRP3KO mice (Supplementary Figures 2A–E). However, there was a trend for more IL-1β (Supplementary Figure 2A) and TNF-α (Supplementary Figure 2B) in bacteremic PGLYRP3KO compared to wt mice.

### PGLYRP3 Does Not Influence *S. pneumoniae*-Dependent Cell Recruitment

A pro-inflammatory cytokine response usually leads to an enhanced cell recruitment to the site of infection. Several cell types of the innate and adaptive immune system were analyzed in the lungs of infected and uninfected wt and PGLYRP3KO mice to determine possible differences between the resident and recruited cells.

First, we analyzed the total amount of leukocytes in uninfected and infected wt and PGLYRP3KO mice 48 h p.i. to check if there was a difference due to leukocytosis. The results showed, that there are no differences between the varying conditions in this analysis (**Figure 4A**) and therefore, relative abundance will be given for the further analysis of cell populations.

When analyzing cell populations in the lung, infected wt mice showed higher percentages of granulocytes (**Figure 4B**), DCs (**Figure 4C**) and NK cells (**Figure 4H**) and lower percentages of CD4^+^ (**Figure 4D**), CD8^+^ (**Figure 4E**) and B cells (**Figure 4F**) compared to PBS-treated mice. This was significant for DCs, CD4^+^, CD8^+^, and B cells. No differences were seen in AMs (**Figure 4G**) and γδ-T cells (**Figure 4I**). PGLYRP3KO mice showed the same patterns but increase was not significant for DCs (**Figure 4C**) and significant for granulocytes (**Figure 4B**). Comparing infected wt and PGLYRP3KO mice, there were no significant differences between these groups. However, there was a trend toward higher percentages of granulocytes in infected PGLYRP3KO compared to wt mice (**Figure 4B**).

### Lung Histology Was Not Altered in PGLYRP3KO Mice

Lung histology was performed 48 h after infection with 10^5^ CFUs *S. pneumoniae* (NCTC 7978) per mouse. The sections were formalin-fixed and stained with HE. Histological examination revealed, as expected, immune cell recruitment and tissue damage in infected mice but independent of the genotype (**Figure 5A**). For quantification of affected/inflamed lung areas, the lungs were digitally analyzed using Genie histology pattern recognition software (**Figure 5B**). There were no differences between the two genotypes, neither in severity nor in expansion of lung lesions. This is in line with our findings that wt and PGLYRP3KO do not differ significantly in CFU count, cytokine response or cell recruitment.

### PGLYRP3KO Mice Do Not Differ in Clinical Symptoms or Survival Compared to the wt Mice during Pneumococcal Infection

To assess the effect of PGLYRP3 on the progression and the final outcome of pneumonia, infected wt and PGLYRP3KO mice were studied over a period of 10 days. The animals were infected with 10^5^ CFUs of *S. pneumoniae* per mouse and were monitored twice a day to assess clinical manifestations and if necessary, they were euthanized at fixed human endpoints.

Most of the wt and PGLYRP3KO mice had to be euthanized between days 4 to 6 after infection. Overall, around 25% of the mice survived the 10-day surveillance with no differences between wt and PGLYRP3KO mice (**Figure 6A**). Furthermore, no differences could be observed in the temperature curve (**Figure 6B**) and the loss of the body weight (**Figure 6C**) when comparing the progression of these clinical markers in wt and PGLYRP3KO animals. After 10 days, all remaining animals showed no clinical symptoms and were declared as healthy survivors.

## Discussion

Since there is an increasing incidence of multi-resistant pathogens in general and also in *S. pneumoniae* strains, alternative treatment strategies are of utmost importance (Spellberg et al., 2015). PGRPs have been described to be at least bacteriostatic or even bactericidal against a variety of different Gram-positive and Gram-negative pathogens (Lu et al., 2006).

Furthermore, it has been reported that mouse PGLYRP3 is constitutively expressed in lung tissues (Mathur et al., 2004) and has beneficial effects in *S. aureus*-induced lung infections (Lu et al., 2006), but to the best of our knowledge, there are no reports illustrating the activity of PGLYRPs against *S. pneumoniae* and unfortunately, there is also no commercially available murine PGLYRP3 to address this question *in vitro*. Therefore, we analyzed the expression patterns of PGLYRP3 in murine AECs, AMs, and bone marrow derived neutrophils (PMNs) *in vitro* and investigated the role of PGLYRP3 in an *in vivo* pneumococcal pneumonia model using PGLYRP3KO mice.

Our data demonstrated that PGLYRP3 is expressed in AECs, AMs, and PMNs. This falls in line with other studies, which demonstrated that PGLYRP3 is expressed in mouse hematopoietic cells, epidermal and lung tissue and epithelial cells of different origin (Mathur et al., 2004; Uehara et al., 2007; Ma et al., 2010). The induction of PGLYRP3 in human oral and intestinal epithelial cells can be achieved *via* NOD1/NOD2 (Uehara et al., 2005) or TLR2 ligands (Uehara et al., 2007; Ma et al., 2010; Zenhom et al., 2011).

In contrast to our expectations, PGLYRP3 was not altered by *S. pneumoniae* in lung epithelial cells, but is highly induced in phagocytic cells of both lung and bone marrow origin. In PMNs there was a significant increase of PGLYRP3 by more than 60-fold and this was even more pronounced in AMs with a more than 350-fold induction following the stimulation with *S. pneumoniae*. Since *S. pneumoniae* is also recognized by NOD2 (Opitz et al., 2004) and TLR2 (Schröder et al., 2003) the induction of PGLYRP3 in AMs and PMNs could be mediated by these or other PRRs such as TLR9 which generally recognizes CpG motifs in bacterial DNA (Gürtler and Bowie, 2013) and pneumococcal DNA (Koppe et al., 2012b; Zahlten et al., 2013). There is also a report that suggests the induction of PGLYRP3 in gut epithelial cells by the peroxisome proliferation-activated receptor gamma (PPARγ) (Zenhom et al., 2011). Since *S. pneumoniae* can release extracellular membrane-derived vesicles which could include some potential PPARγ activating fatty acids (Olaya-Abril et al., 2014), the activation of PPARγ could also be a possible path for gene induction. PGLYRP3 is reported to be bacteriostatic or bactericidal against a variety of different bacteria *in vitro* including *B. cereus, B. subtilis, L. monocytogenes, S. aureus, Salmonella enterica, S. pyogenes*, and *E. coli* (Lu et al., 2006; Wang et al., 2007).

Because of this high induction of PGLYRP3 in professional phagocytes and recognition of these bacteria by the immune system, we expected to see a higher bacterial load in PGLYRP3KO mice. Unexpectedly, we did not observe any differences in the bacterial load isolated from the lungs of the PGLYRP3KO mice in comparison to the wt mice 12 or 48 h after pneumococcal infection. Only a tendency to a higher bacterial load in the blood and spleens of the PGLYRP3KO mice compared to the wt mice was found 48 h after infection. Thus, PGLYRP3 seems to have no impact concerning the bacterial clearance of *S. pneumoniae* within the lung but it might have a minor antimicrobial function in the case of sepsis due to pneumococci.

The application of recombinant human PGLYRP3 in a *S. aureus* lung infection mouse model reduced the bacterial load significantly (Lu et al., 2006). To the best of our knowledge, it is not known how much PGLYRP3 is produced in uninfected or infected human or mouse lung tissue. In the previously mentioned publication, they pretreated the mice intranasal with 4.5 μg of human recombinant PGLYRP3 followed by the bacterial challenge. The bacterial load was measured 4 h post infection within the lungs. Since Lu et al. (2006) infected the mice 15 min after treatment with rPGLYRP3 *via* the same route (intranasally), it is possible that the bacteria were already killed before they reached the lung. Another reason could be a difference between human and mouse PGLYRP3 [human rPGLYRP3 in Lu et al. (2006) *vs.* mouse PGLYRP3 in this study]. Until now, it is unknown whether human PGLYRP3 is bactericidal against *S. pneumoniae* or if mouse PGLYRP3 has the same antimicrobial efficiency and range like human PGLYRP3. This should be investigated in further studies.

It has been implied that the regular bacterial flora have developed evasion mechanisms and resistance against AMPs or proteins such as PGLYRPs present in the skin, eyes and mucous membranes and can therefore colonize these areas (Lu et al., 2006). *S. pneumoniae* is an asymptomatic commensal of the nasopharynx and an opportunistic pathogen (Kadioglu et al., 2008) and may have developed strategies like other bacteria of the microbial flora in the upper respiratory tract and intestine to evade antimicrobial effects (e.g., by PGLYRPs). One of these strategies could be the de-*N*-glycosylation of the PGLYRPs. *N*-glycosylation is necessary for the accurate function of the PGLYRPs. Lu et al. (2006) showed that after the enzymatic de-*N*-glycosylation with PNGaseF, the antibacterial activity of the PGLYRPs was completely abolished (Lu et al., 2006). It is known that *S. pneumoniae* has the ability to produce de-glycosylation enzymes, which are active *in vivo* (Kadioglu et al., 2008; Sjögren and Collin, 2014; Dukhanina et al., 2015). Due to diverse glycosylation patterns it is unknown if these enzymes produced by *S. pneumoniae* can or cannot inactivate PGLYRPs. Another mechanism for how *S. pneumoniae* might inhibit PGLYRP function was published recently. In this study, the authors demonstrated that a genomic island (GI)-type-IVC secretion system-dependent release of streptococcal SP1 protein interacts and thereby inhibits PGLYRP1 function (Wang et al., 2017).

Thus, if PGLYRPs could be inactivated upon infection with *S. pneumoniae*, a knockout model would only show, if any, slight changes in the overall outcome even though there could be differences in the gene or protein expression of the PGLYRPs.

Analyzing the inflammatory response in the lungs, we saw, as expected, an induction of pro- (IL-1β, TNF-α, KC, IL-6) and anti-inflammatory (IL-10) chemo- and cytokines following infection with *S. pneumoniae*. There was only a tendency toward enhanced TNF-α secretion between wt and PGLYRP3KO in pneumococcus-infected mice lungs. The higher amounts of pro-inflammatory cytokine response in the knockout animals would align to the findings of Zenhom et al. (2011). Zenhom et al. (2011) pointed out, that the induction of PGLYRP3 lead to an induction of IκBα protein, which in turn lead to suppression of NF-κB activation and an overall anti-inflammatory phenotype. Because we did not see any significant differences in the inflammatory response in the lungs between wt and PGLYRP3KO mice *in vivo*, there could be mechanisms by which *S. pneumoniae* alters PGLYRP3-activated immune functions. It was previously shown, that PGLYRP1 also needs *N*-glycosylation to be bactericidal (Lu et al., 2006). However, other immune functions of PGLYRP1 remain unaffected as previously shown (Dukhanina et al., 2015; Yashin et al., 2015). In these studies, they expressed recombinant PGLYRP1 in *E. coli*, an expression system that is not able to glycosylate proteins. Nevertheless, they still demonstrated chemoattractant and cytotoxic properties of PGLYRP1 (Dukhanina et al., 2015; Yashin et al., 2015).

Consistent with our other results, we saw no significant differences in the cell recruitment by multi-color flow cytometry and the lung histopathology did not reveal any clear changes in the affected area, grade of inflammation, influx of immune cells or localization of morphological lesions between uninfected or infected wt *vs.* PGLYRP3KO animals. Nevertheless, there was a higher percentage of neutrophils and dendritic cells and a lower percentage of T and B cells upon infection in both groups. This fall in line with the activation of the innate immune system with the recruitment of neutrophils and dendritic cells to the side of infection. Noteworthy was a doubling of neutrophils upon infection of wt mice (9.9% *vs.* 20.4%; control *vs.* infection, respectively) in the lung and nearly tripling in the PGLYRP3KO mice (9.9% *vs.* 27.9%; control *vs.* infection, respectively), but this occurred without reaching statistical significance. These differences were not observed in the histopathological analysis. The inconsistent results between the flow cytometry and histopathological analyses may be explained by the limitations of the qualitative and semi-quantitative evaluation of histopathological samples. During these analyses certain areas of the lung may be overlooked where flow cytometry analysis will evaluate the cells in the entire lung.

Finally, there were no differences in the main outcome, the overall survival of infected animals or the onset of quantitative clinical symptoms, namely body temperature and weight loss. Moreover, we did not observe significant differences in the bacterial burden between wt and PGLYRP3-deficient mice at a very early (12 h p.i.) and a more progressed state of infection (48 h p.i.). Nevertheless, we cannot fully exclude, that a change in the infection dose may have shown differences in these parameters due to PGLYRP3-deficiency.

In summary, we performed an infection study with PGLYRP3KO mice with a pathogenic *S. pneumoniae* type 3 strain. Within this study, we analyzed the bacterial burden in the lung and periphery, the inflammatory chemo- and cytokine response, the cell recruitment and histopathology in the lung as well as the clinical manifestations and the overall survival of infected animals. In all these experiments we did not see any significant differences between wt and PGLYRP3KO mice. Nevertheless, we saw differences in the gene expression of PGLYRP3 upon infection of isolated primary AMs and PMNs. In the AMs we saw a 66-fold and in neutrophils a 373-fold induction of the PGLYRP3 gene expression. Therefore, we conclude that the host responds to the infection with *S. pneumoniae* by induction of PGLYRP3, but this pathogen might have evasion mechanisms like glycosidases, which may neglect at least the direct antibacterial effects of PGLYRP3. To prove this hypothesis, studies on the interaction of PGLYRPs with *S. pneumoniae* have to be carried out. These studies will lead to a better understanding of pneumococcal evasion strategies and shed light on the interaction of pneumococci with the innate immune system.

## Author Contributions

AS, AD, CC, NB, SP, and KD performed the experiments, AS, AD, CC, NB, SP, KD, and AG did formal analysis of the data. AS, AD, and JZ wrote the original draft, AD, NB, HH, SP, KD, AG, NS, and JZ reviewed and edited the manuscript. SA and PN did conceptualization work. SA, PN, NS, and JZ supervised the work. SA, PN, and NS did funding acquisition. NS contributed resources and JZ did the project administration.

## Conflict of Interest Statement

The authors declare that the research was conducted in the absence of any commercial or financial relationships that could be construed as a potential conflict of interest.
