# Supplementary material for: Peptidoglycan Recognition Protein 3 Does Not Alter the Outcome of Pneumococcal Pneumonia in Mice
Source: Front Microbiol. 2018 Feb 1;9:103. doi: 10.3389/fmicb.2018.00103 (PMC5799233; doi:10.3389/fmicb.2018.00103)
Supplement: Supplementary file 1 [file Image_1.PDF]

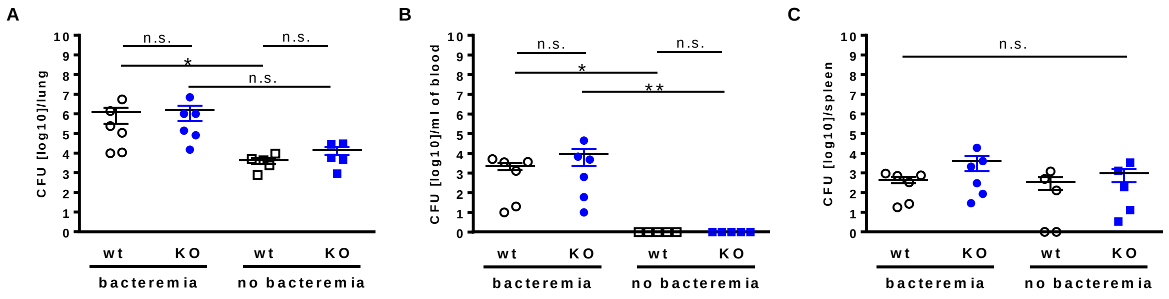

Supplemental Figure 1: The loss of PGLYRP3 did not affect the bacterial load when analyzed separately in bacteremic and non-bacteremic mice.

Mice were infected intranasally with 10^5 CFUs of the *S. pneumoniae* strain NCTC 7978 per mouse. The lungs (A), the blood (B) and the spleens (C) were analyzed 48 h after infection for the bacterial load in wt and PGLYRP3KO mice separately for bacteremic and non-bacteremic mice. Five to six mice per group were analyzed (mean ± SEM); Kruskal-Wallis with Dunn's multiple comparison test: * $p < 0.05$; * $p < 0.01$; ns: not significant.
