# Supplementary material for: Peptidoglycan Recognition Protein 3 Does Not Alter the Outcome of Pneumococcal Pneumonia in Mice
Source: Front Microbiol. 2018 Feb 1;9:103. doi: 10.3389/fmicb.2018.00103 (PMC5799233; doi:10.3389/fmicb.2018.00103)
Supplement: Supplementary file 2 [file Image_2.pdf]

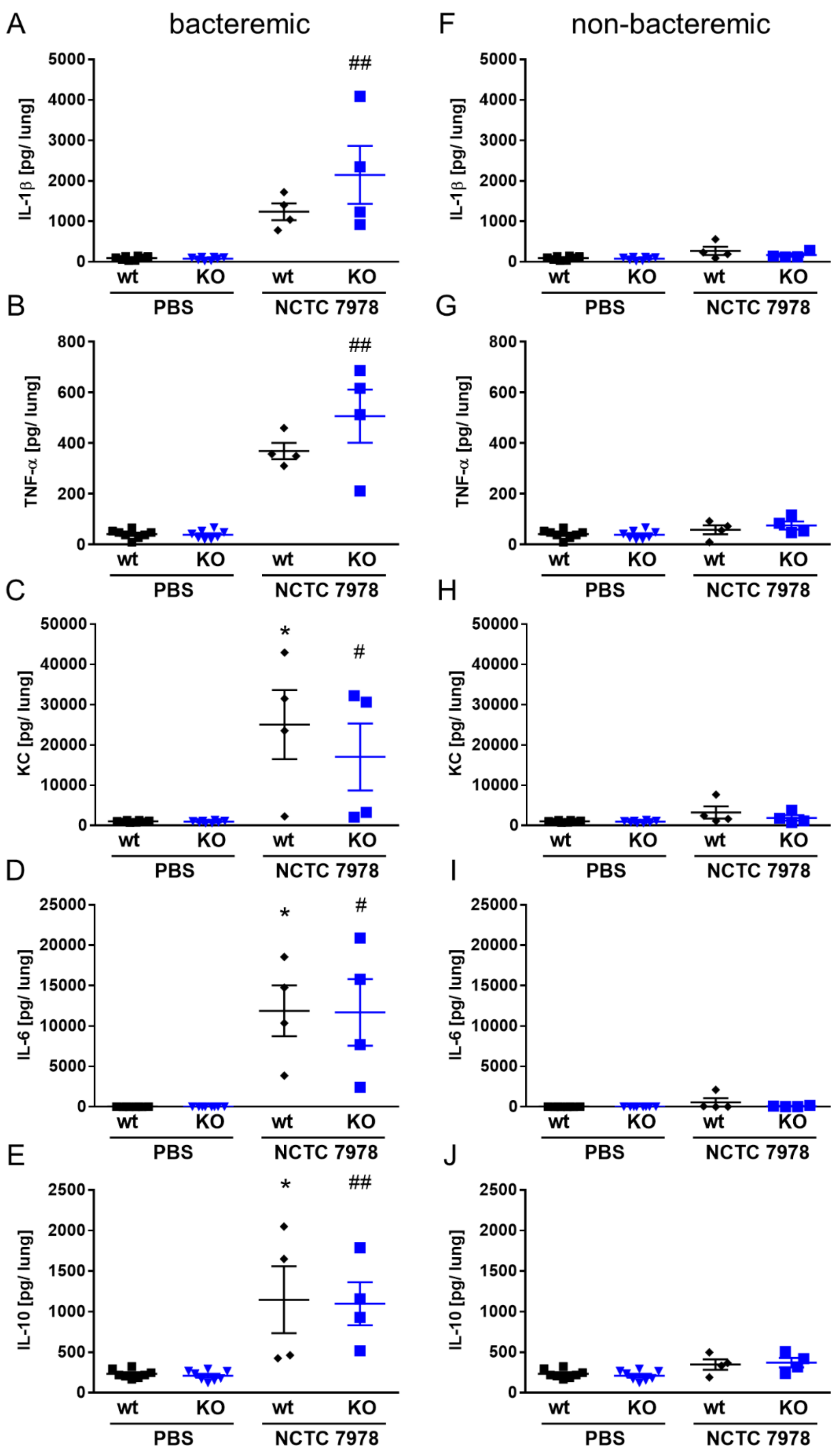

Supplemental Figure 2: Cytokine response in the lung of bacteremic and non-bacteremic wt and PGLYRP3KO mice. The *S. pneumoniae*-dependent cytokine response was analyzed 48 h post infection (10^5 CFUs/mouse, NCTC 7978) in lung homogenates of bacteremic (A-E) and non-bacteremic mice (F-J). The production of IL-1β (A,F), TNF-α (B,G), KC (C,H), IL-6 (D,I) and IL-10 (E,J) were quantified by ELISA. Four mice per group (mean ± SEM); Kruskal-Wallis with Dunn's multiple comparison test: uninfected *vs.* infected wt: * $p < 0.05$ and uninfected *vs.* infected PGLYRP3KO: # $p < 0.05$; ## $p < 0.01$.
